# Erythematous, pruritic and indurated plaques following treatment for melanoma

**DOI:** 10.1093/skinhd/vzae004

**Published:** 2025-11-13

**Authors:** Hareni Srenathan, Jing Gao, Eva Kolson Kokohaare, Ferina Ismail

**Affiliations:** Department of Dermatology, Royal Free London NHS Foundation Trust, London, UK; Department of Dermatology, Royal Free London NHS Foundation Trust, London, UK; Department of Histopathology, Royal Free London NHS Foundation Trust, London, UK; Department of Dermatology, Royal Free London NHS Foundation Trust, London, UK

## Abstract

A 35-year-old man presented with two itchy, nontender, indurated erythematous plaques on his forehead and upper back. Three months prior, he had completed a 12-month course of adjuvant pembrolizumab for stage IIIC melanoma. Clinical and imaging surveillance revealed no evidence of melanoma recurrence of his left cheek. Incisional biopsies of both plaques with immunohistochemistry favoured a CD4^+^ reactive lymphoid process in keeping with cutaneous pseudolymphoma (CPL), secondary to pembrolizumab, an immune checkpoint inhibitor (ICI). The conspicuous, pruritic forehead plaque was unresponsive to clobetasol proprionate under occlusion. A single course of intralesional triamcinolone acetonide 10 mg L^−1^ resulted in significant improvement within days, with subsequent complete resolution in a month. The plaque on the back resolved spontaneously after 6 months. Drug-induced CPL describes an adverse cutaneous drug reaction mimicking B- or T-cell lymphomas clinically and/or histologically. It has been described with anticonvulsants, antidepressants and biologic agents, often with resolution on cessation of the responsible drug. It has been proposed that the drugs affect immune surveillance, leading to an abnormal cutaneous lymphocyte response. As with the other adverse effects of immunotherapy, it is likely that CPL with ICIs is due to an iatrogenic immune dysregulation leading to T-cell overactivity. We highlight the successful use of intralesional steroid as treatment for drug-induced CPL with an ICI. It is important to recognize CPL as an adverse cutaneous effect of immunotherapy, and that it can present after completing treatment due to an ongoing immune response.

## Teaser

We present a case of drug-induced cutaneous pseudolymphoma (CPL) secondary to pembrolizumab and highlight the successful use of intralesional steroid as a treatment option. It is important to recognize CPL as an adverse cutaneous effect of immunotherapy and that it can present after completing treatment due to an ongoing immune response.

What is already known about this topic?

- Cutaneous pseudolymphoma (CPL) is a reactive process with clinical and/or histological features mimicking cutaneous lymphoma.
- Drug-induced CPL is a common subtype of induced CPL with monoclonal antibodies contributing to a significant proportion of these cases.

What does this study add?

- Drug-induced CPL can have a delayed presentation, particularly with immunotherapy, due to its ongoing immune response.
- Drug-induced CPL is steroid-responsive and treatment options include intralesional steroid for cases that are symptomatic and/or involve prominent areas.

Drug-induced cutaneous pseudolymphoma (CPL) describes an adverse cutaneous drug reaction mimicking B- or T-cell lymphomas clinically and/or histologically. It has been described with anticonvulsants, antidepressants and biologic agents, often with resolution on cessation of the responsible drug. It has been proposed that the drugs affect immune surveillance, leading to an abnormal cutaneous lymphocyte response. As with the other adverse effects of immunotherapy, it is likely that CPL with immune checkpoint inhibitors (ICIs) is due to an iatrogenic immune dysregulation leading to T-cell overactivity. We highlight the successful use of intralesional steroid as treatment for drug-induced CPL with an ICI. It is important to recognize CPL as an adverse cutaneous effect of immunotherapy, and that it can present after completing treatment due to an ongoing immune response.

## Case report

### Clinical findings

**Figure 1:**
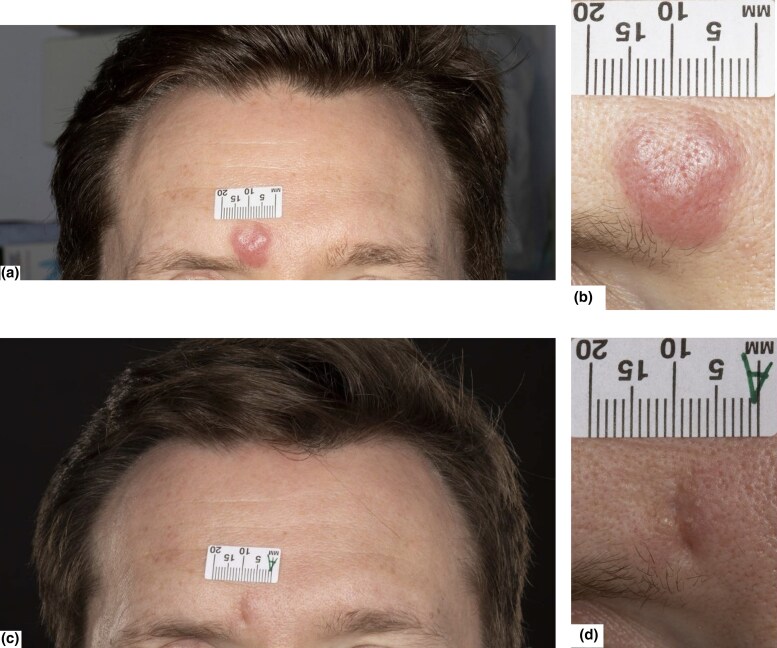
Clinical photos of an erythematous, indurated forehead lesion. (a, b) At presentation. (c, d) Resolution following intralesional triamcinolone.

A 35-year-old man presented with a 7-week history of two itchy, nontender, indurated erythematous plaques, on his forehead (Figure 1a and b) and on his upper back, with no discriminatory clinical features. He was systemically well and had no concurrent medications.

Three months prior, he had completed a 12-month course of adjuvant pembrolizumab for stage IIIC melanoma. The primary melanoma had been excised from his left cheek, with involvement of left parotid and supraclavicular lymph nodes, treated with clearance via superficial parotidectomy and left neck dissection. Clinical and imaging surveillance revealed no evidence of melanoma recurrence.

The plaque on the forehead was particularly pruritic and prominent with no response to clobetasol propionate under occlusion. A single dose of 0.6 mL intralesional triamcinolone acetonide 10 mg mL^−1^ resulted in significant improvement within days, with subsequent complete resolution within 1 month (Figure 1c and d). The plaque on the back resolved spontaneously after 6 months.

### Histopathological findings

**Figure 2:**
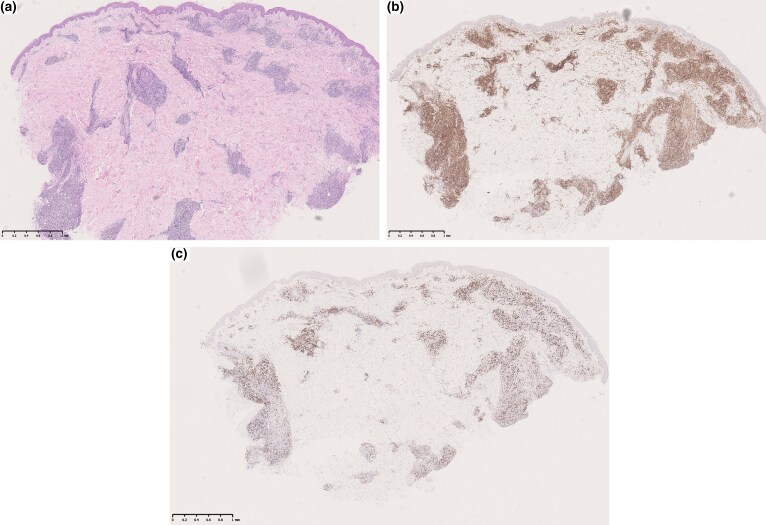
Histology. (a) Haemotoxylin and eosin, ×20 magnification. Superficial and deep dermal perivascular and periadnexal infiltrate of small and medium-sized lymphoid cells. Immunohistochemistry staining favoured a (b) CD4^+^ phenotype when compared with (c) CD8 stain.

Incisional biopsies of the plaques on his back and forehead both demonstrated a superficial and deep dermal perivascular and periadnexal infiltrate of small and medium-sized lymphoid cells (Figure 2a). There was no epidermotropism and B- and T-cell clonality studies were negative. Morphological appearances and immunophenotype favoured a CD4^+^-reactive lymphoid process (Figure 2b and c).

### Diagnosis

Given these clinical and histological findings, a diagnosis of drug-induced CPL secondary to pembrolizumab was made.

## Discussion

CPL is a reactive lymphoproliferative process that mimics B- or T-cell lymphomas clinically and/or histologically.^1^ Predominance may be T cell, B cell or mixed and while the majority of cases are idiopathic, common triggers include contact dermatitis and foreign agents, e.g. tattoo dyes, infection, arthropod bites, photosensitivity and drugs.^2^

CPL may present as papules, infiltrative plaques and nodules or erythroderma, as seen with cutaneous lymphoma. Histological features that may support a diagnosis of CPL and differentiate it from cutaneous lymphoma include the absence of epidermotropism; the predominance of small or medium-sized lymphocytes with no significant number of large, atypical cells; no significant loss of lymphoid markers on immunohistochemistry; and negative clonality studies.

With no pathognomonic diagnostic features, a clear clinical history with histology and relevant specialist diagnostic tests are required to make the correct diagnosis of CPL. Progression to cutaneous lymphoma is rare but has been described.^3^

Drug-induced CPL, a common subtype of induced CPL, is usually T-cell-dominant and has been described with anticonvulsants, antidepressants and biologic agents, often with resolution on cessation of the responsible drug.^4^ It has been proposed that the drugs affect immunosurveillance, leading to an abnormal cutaneous lymphocyte response mimicking cutaneous lymphoma.

Pembrolizumab, the likely causative agent in our patient, is an ICI, which blocks programmed cell death protein 1 (PD-1) and boosts the host anti-tumour T-cell response.^5^ Drug-induced CPL with other ICIs has been described. A recent case report^6^ described CPL secondary to ipilimumab and nivolumab in a 38-year-old man presenting with erythematous plaques on the hands and face, clinically similar to that of our patient. The lesions appeared after the fourth cycle of the combination treatment and he was otherwise systemically well. The plaques responded to treatment with prednisolone. As with the other adverse effects of immunotherapy, it is likely that the drug-induced CPL with ICIs is due to an iatrogenic immune dysregulation leading to T-cell overactivity.

A systematic review of drug-induced CPL identified that monoclonal antibodies, including ipilimumab, secukinumab, tocilizumab, infliximab, adalimumab and cetuximab, contribute to a significant proportion of these cases.^4^ As with our patient, a T-cell predominance and longer interval time to presentation was associated with this drug category. Immunotherapies in melanoma have been identified to have an immunomodulating effect after cessation of treatment and have delayed immune-related adverse events,^7^ which may explain the delayed onset of plaques in our patient. CD30 expression was also observed to be lowest with drug-induced CPL caused by monoclonal antibodies.

While it is difficult to determine causality in a case report, no other trigger for CPL was identified in the clinical history or in the examination of our patient, implicating pembrolizumab as the most probable causative agent. No new plaques appeared following this episode, suggesting that another ongoing trigger is less likely. Drug-induced CPL is steroid-­responsive,^2^ as seen in our patient with the rapid clearance of the forehead plaque following intralesional steroid. Our case report adds to the current literature describing drug-induced CPL with an ICI and larger studies are required to understand the association with pembrolizumab.

With clinical and histological features similar to that of primary cutaneous lymphoma, it is important to differentiate drug-induced CPL, which is steroid-responsive. Treatment may be considered in symptomatic cases, with pruritus being the commonest feature^4^ or when lesions are in visible sites. Taking a comprehensive drug history and interpreting the histology with appropriate immunohistochemistry is essential to diagnosis and management.

We highlight the successful use of intralesional steroid as a treatment option for drug-induced CPL with an ICI. This case adds to the importance of recognizing CPL as an adverse cutaneous effect of immunotherapy and that it can present after completing treatment, due to an ongoing immune response seen with this class of drugs.

## Data Availability

The data underlying this article will be shared on reasonable request to the corresponding author.
